# Single-pulse ultrafast real-time simultaneous planar imaging of femtosecond laser-nanoparticle dynamics in flames

**DOI:** 10.1038/s41377-024-01588-x

**Published:** 2024-08-29

**Authors:** Yogeshwar Nath Mishra, Peng Wang, Florian J. Bauer, Murthy S. Gudipati, Lihong V. Wang

**Affiliations:** 1 Caltech Optical Imaging Laboratory, Andrew and Peggy Cheng Department of Medical Engineering, Department of Electrical Engineering, California Institute of Technology, 1200 East California Boulevard, Mail Code 138-78, Pasadena, CA 91125 USA; 2 Science Division, Jet Propulsion Laboratory, California Institute of Technology, 4800 Oak Grove Drive, Pasadena, CA 91109 USA; 3 Lehrstuhl für Technische Thermodynamik (LTT) and Erlangen Graduate School in Advanced Optical Technologies (SAOT), Universität Erlangen-Nürnberg, Erlangen, 91058 Germany

**Keywords:** Imaging and sensing, Nanoparticles, Ultrafast photonics

## Abstract

The creation of carbonaceous nanoparticles and their dynamics in hydrocarbon flames are still debated in environmental, combustion, and material sciences. In this study, we introduce single-pulse femtosecond laser sheet-compressed ultrafast photography (fsLS-CUP), an ultrafast imaging technique specifically designed to shed light on and capture ultrafast dynamics stemming from interactions between femtosecond lasers and nanoparticles in flames in a single-shot. fsLS-CUP enables the first-time real-time billion frames-per-second (Gfps) simultaneous two-dimensional (2D) imaging of laser-induced fluorescence (LIF) and laser-induced heating (LIH) that are originated from polycyclic aromatic hydrocarbons (PAHs) and soot particles, respectively. Furthermore, fsLS-CUP provides the real-time spatiotemporal map of femtosecond laser-soot interaction as elastic light scattering (ELS) at an astonishing 250 Gfps. In contrast to existing single-shot ultrafast imaging approaches, which are limited to millions of frames per second only and require multiple laser pulses, our method employs only a single pulse and captures the entire dynamics of laser-induced signals at hundreds of Gfps. Using a single pulse does not change the optical properties of nanoparticles for a following pulse, thus allowing reliable spatiotemporal mapping. Moreover, we found that particle inception and growth are derived from precursors. In essence, as an imaging modality, fsLS-CUP offers ultrafast 2D diagnostics, contributing to the fundamental understanding of nanoparticle’s inception and broader applications across different fields, such as material science and biomedical engineering.

## Abstract (web summary)

Femtosecond Laser-sheet Compressed Ultrafast Photography (fsLS-CUP) is the world’s fastest real-time planar imaging camera (250 billion fps), enabling nanosecond observation of laser-nanoparticle interactions and hydrocarbon dynamics in flames simultaneously.

## Graphical abstract

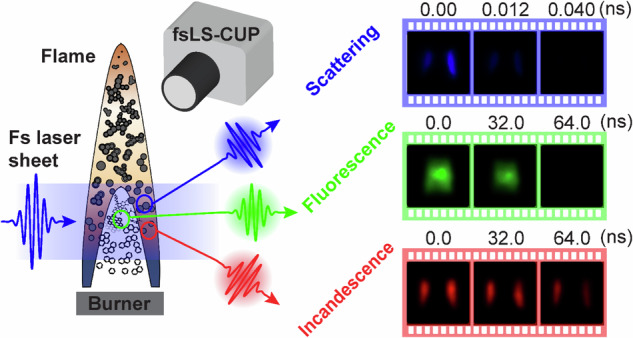

## Introduction

Nanosized-soot particles, caused by the incomplete combustion of hydrocarbon flames^1^, are of paramount importance due to their significant impact on our environment^2^, health^3^, and their potential use as nanomaterials^4^. Nanosecond lasers have been extensively employed to characterize both soot nanoparticles and their precursors, known as polycyclic aromatic hydrocarbons (PAHs)^5–8^. Laser-induced fluorescence (LIF) and laser-induced incandescence (LII) are popular methods to investigate PAHs and soot, respectively^5^. LIF involves the emission of photons when laser light excites valence electrons in PAH molecules, while LII results from the thermal radiation emitted by heated soot particles upon laser irradiation. Various properties of soot particles, such as their primary particle size, aggregate size, and soot volume fraction, are measured through time-resolved LII, laser extinction, and elastic light scattering (ELS) experiments primarily using the nanosecond lasers^8^. The duration of LII signals emitted by soot particles can range from tens of nanoseconds to hundreds of nanoseconds, and in high-pressure conditions, they can be as short as a few nanoseconds (ns)^9^. PAHs, on the other hand, exhibit LIF lifetimes ranging from a few ns down to tens or hundreds of picoseconds (ps), decreasing as the flame temperature rises^5,10^. Beside the diagnostic challenge to capture these fast signals, differences between nanosecond and picosecond LII^10^, the sizes of these particles and molecular clusters^11^ are important quantities to understand the formation and growth dynamics.

To gain a deeper understanding of nanoparticles formation phenomena in flames and resolve their dynamics, ultrafast single-shot two-dimensional (2D) images of PAHs, soot, and their properties are required. While earlier efforts employed multiple laser pulses or clusters of nanosecond lasers for ultrafast imaging of nanoparticles at millions of frames per second^12,13^, it remains a challenge till now to develop a single-pulse real-time 2D imaging system operating at billion-frames-per-second (Gfps). A single pulse-based method is preferred because the multiple laser pulses change the optical properties of nanoparticles in the flame. Further, a single-pulse single-shot ultrafast imaging system can film the ultrafast physical and chemical processes governing PAH growth, soot inception, and soot formation in turbulent flames. Thanks to the compressed ultrafast photography (CUP) technology, it is possible to capture optical phenomena at unprecedented imaging speeds ranging from billions to trillions of frames per second with a maximum sequence depth of up to 1000 frames^14–16^. Recently, laser-sheet compressed ultrafast photography (LS-CUP) was introduced for single-pulse, single-shot planar imaging to record the ultrafast dynamics of laser interactions with nanoparticles in laminar diffusion flames^17^. This technique, for the first time, enabled the spatiotemporal recording of optical signals, including LIF, LII, and ELS from nanoparticles at imaging speeds of up to tens of Gfps in sequential measurements. Despite nanosecond time resolution being adequate to sequentially resolve PAHs and soot dynamics, simultaneous observation has not been achieved yet in a single-pulse excitation. Observing PAHs and soot simultaneously at higher temporal resolution could reveal crucial insights, such as the onset of soot formation beginning with the creation of PAH precursors and femtosecond laser-nanoparticle interactions.

Femtosecond lasers have emerged as invaluable tools for studying soot particles and other species in combustion processes^18^. By employing femtosecond lasers, researchers can gain deeper insights into the fundamental mechanisms of creation of carbonaceous nanoparticles in flames. For example, to determine the size of PAHs, femtosecond lasers and streak cameras have been employed, capitalizing on their fluorescence polarization anisotropy^19^. Recent research using femtosecond pump-probe spectroscopy has suggested that soot nanoparticles undergo ultrafast swelling at around 100 fs and a relatively slow shrinking process at about 1 ps^18^. Furthermore, femtosecond lasers can be utilized as an excitation source in combustion diagnostics. It is accomplished by employing either single-photon resonance or nonlinear multiphoton excitations to induce fluorescence emissions from combustion intermediates, encompassing species and radicals such as O, H, OH, NH, and CH^20^. It’s worth noting that femtosecond filaments have been documented for achieving multispecies excitation within flames. However, all these femtosecond laser experiments are primarily one-dimensional (1D) and their results are averaged over several shots.

Inspired by the aspiration for real-time simultaneous planar observation of PAHs and soot particles using a single pulse femtosecond ultra-violet (UV) wavelength excitation, in our research, we present a pioneering technique called femtosecond laser sheet-compressed ultrafast photography (fsLS-CUP), meticulously engineered to capture 2D, single-pulse, single-shot, ultrafast dynamics resulting from the interplay between femtosecond laser and flames. We probed well-known laminar flames, ideal for soot characterization due to their stability, axisymmetric geometry, and repeatability. fsLS-CUP allows for the real-time simultaneous monitoring of laser-induced fluorescence arising from PAHs, and thermal radiation emitted from soot particles. We also applied fsLS-CUP to image elastic light scattering associated with soot particles with a sub 20 ps temporal resolution. The spectral properties and duration of the signal produced through femtosecond laser-induced processes closely resemble the laser-induced incandescence (LII) observed in soot particles when employing a nanosecond laser pulse. Consequently, the signal induced by femtosecond lasers, termed laser-induced heating in our study, could potentially serve as the initial evidence of LII, as reported in our work. Finally, while the approach can potentially extend to turbulent cases, we focus on diagnostic development.

## Results

### Experimental setup

**Fig. 1:**
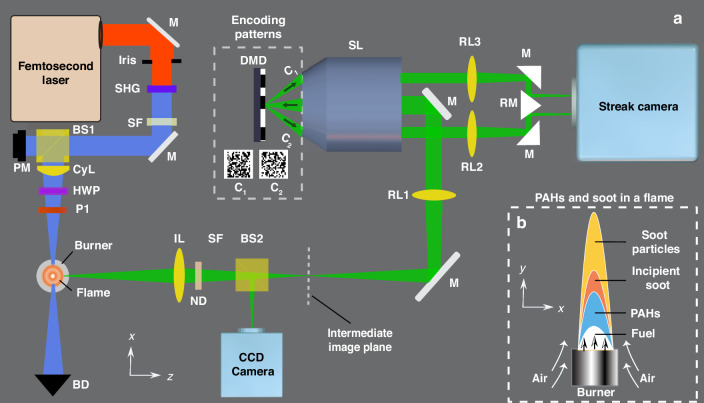
fsLS-CUP for time-resolved 2D imaging of femtosecond laser-induced flame signals. **a** A top-view schematic of the fsLS-CUP setup is depicted, implemented with a femtosecond laser, a CCD camera, a DMD, a streak camera, and various optical components. The top left box corresponds to the image encoding using the DMD, where two complementary copies are formed. **b** A schematic side view of the flame, highlighting the locations of PAHs, incipient particles, and soot particles in different regions of a sooty laminar flame

Figure 1 shows the schematic of the fsLS-CUP’s optical setup (Fig. 1a) and identification of PAHs and soot particles in flame (Fig. 1b). A femtosecond laser (Ti:Saphire) of 800 nm wavelength is utilized as the laser source, featuring a 70 fs pulse duration at full-width-at-half-maximum (FWHM), a repetition rate of 500 Hz, and an approximate beam diameter of 10 mm. A second harmonic generation crystal (BBO) and a short-pass filter is used to produce a femtosecond pulse of 400 nm wavelength, utilized for the flame study. The laser beam is then guided through a 90/10 beam splitter (BS1) for the flame study and for laser fluence monitoring. A cylindrical lens with a focal length of 500 mm (CyL) generates a laser sheet with a thickness of approximately 25 µm within a symmetric kerosene flame. After the cylindrical lens, a half-wave plate (HWP) and a linear polarizer (P1) are used for adjusting the laser fluence. The selection of kerosene as the fuel in this study stems from its versatility, as it finds applications ranging from household use to powering combustion engines. The burner is mounted on a three-axis translation stage, allowing the laser sheet to illuminate different positions within the flame. The polarization of the laser sheet is oriented along the $y$-axis. The femtosecond laser-induced flame dynamics are initially captured and imaged onto the intermediate image plane through a pair of 2-inch lenses (L1) and (L2). A 2-inch lens (L3) in combination with a stereoscopic lens assembly (SL) relays the image to the digital micromirror device (DMD) for spatial encoding. The reflected light from the DMD is divided into two beam paths, each masked by complementary encoding patterns C1 and C2 (dashed box). Refer to Supplementary Information for more details regarding the structure of the DMD. An example of an encoding mask displayed on DMD is also given in Fig. S1. The SL, with a numerical aperture of 0.5, can collect both reflected beams and works alongside 1-inch lenses (L4 and L5) to relay the encoded images to the entrance of a streak camera (Hamamatsu, C7700). The streak camera can effectively separate frames from consecutive transient moments by first converting charge-neutral photons to negatively charged electrons and then imparting an ultrafast sweeping voltage. The imaging speed of fsLS-CUP is essentially determined by the slope of the sweeping voltage ramp. More details on the streak camera can be found in Supplementary Information, and its internal construction is shown in Fig. S2. A knife-edge right-angle prism mirror (KRPM) folds the two images so that they can fit side by side at the entrance of the streak camera without overlapping. A 50:50 (R:T) beam splitter (BS2) separates the time-sheared views from the time-unsheared view, which is captured by an external CCD camera (Point Gray). Additionally, there are neutral density filters (ND) and spectral filter (SF) that can be configured as needed to adapt to various imaging scenarios. The transmittances of the bandpass spectral filters used in this work are plotted in Fig. S3. The dashed box (Fig. 1b) in bottom right highlights the locations of PAHs, incipient particles, and soot particles in different regions of a sooty laminar flame. Using fsLS-CUP, we probed the central plane of the flame starting from the origin of the flame to the area above the inception zone. The incident fluence for the experiments is 0.01–0.03 J cm^−2^.

### Real-time simultaneous observation of PAH-LIF and soot-LIH

Real-time two-dimensional observation of combustion processes is rarely documented, making it difficult to study intermediate chemical species and real-time reactions during soot formation and growth with nanosecond and picosecond temporal resolutions. Simultaneously observing PAH molecules and soot particles provides better insights into understanding the formation of initial particles and establishing the intricate relationship between PAHs and soot^21^.

**Fig. 2:**
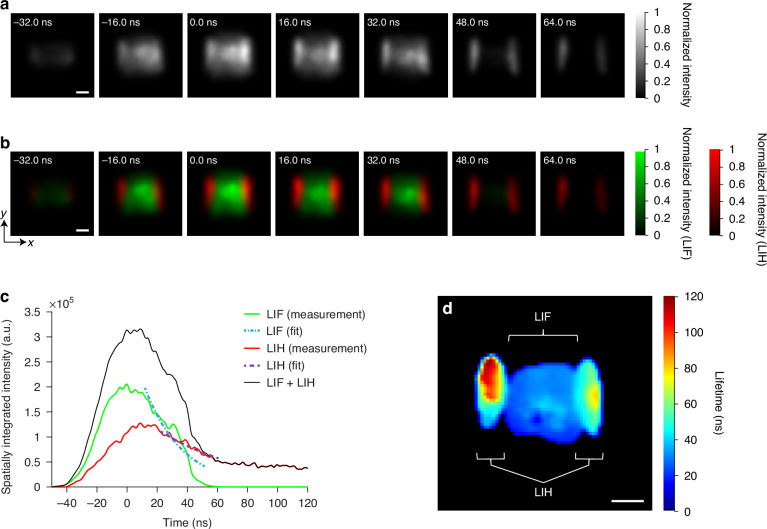
1.25 Gfps real-time simultaneous imaging of laser-induced fluorescence (LIF) of PAH molecules and laser-induced heating (LIH) of soot nanoparticles. Panels **a** and **b** show the grayscale and pseudo-colored 2D snapshot images that capture the rapid time-dependent changes in PAHs and soot using a single femtosecond laser pulse. In **b**, the green and red colormaps represent LIF and LIH signals from PAHs and soot particles, respectively. **c** Spatially integrated intensity of LIF and LIH plotted separately and together. These plots show that PAH-LIF and soot-LIH signals peak at around 0 and 20 ns, respectively. Here, time 0 is defined as the time when the LIF + LIH signal reaches its peak. At about 40 ns, LIF and LIH signals intersect, with LIF reaching nearly half its peak intensity and LIH reaching a similar level. By 60 ns, LIH and both signals (LIF + LIH) equalize, while PAH LIF intensity drops to zero. **d** 2D map of LIF and LIH lifetimes. Scale bars: 2 mm

In Fig. 2, we present a series of images that capture the rapid time-dependent signal after a single femtosecond laser pulse in a flame. We recorded the signals at an imaging speed of 1.25 Gfps with a frame interval of 0.8 ns. The complete sequence can be found in Supplementary Movie S1. Note that there are totally 220 frames acquired in single shot. The LIF and LIH signals were detected within the 430–490 nm spectral band. In Fig. 2a, grayscale images are shown, which reveal that there are two different signals with different decay times. The one with the shorter decay time is located in the center of the flame, while the longer lasting signal surrounds it and is at the edges of the flame. Figure 2b presents pseudo-color-labeled images, with the short signal depicted in green and the longer signal in red. Please refer to Fig. S5 in Supplementary Information about how to computationally separate both signals.

The green signal (pseudo-colored in Fig. 2b) matches with respect to the excitation and emission wavelengths LIF signals of PAH molecules. Further, the location of the signal in the center of the flame depicts the typical behavior of a non-premixed flame with a soot-empty fuel core and PAH distributions surrounding it. The radial profiles of LIF exhibit axial symmetry, consistent with previous time-integrated LIF images of laminar diffusion flames^9,22^. In diffusion flames, a strong correlation between fluorescence intensity and PAH concentration can be expected^22^. Therefore, early time-resolved data in our measurements can serve as a qualitative map of PAH molecule concentrations. In the tens of nanosecond timeframe, PAH lifetimes resemble those observed using nanosecond laser pulses^17^. Our measurements are consistent with the temporally resolved laser-induced emissions at a detected wavelength of 450 nm using 266 nm excitation of a nanosecond laser, near the burner^5^. Nevertheless, here, the PAHs maps are appearing more prominent in the inner core of the flame because of the excitation wavelength is 400 nm in comparison to 532 nm as used in previous work^16^. Using this lower wavelength makes it possible to excite the smaller PAH molecules of only a few aromatic rings^23^, which do not absorb at longer wavelengths like 532 nm^24^.

**Fig. 3:**
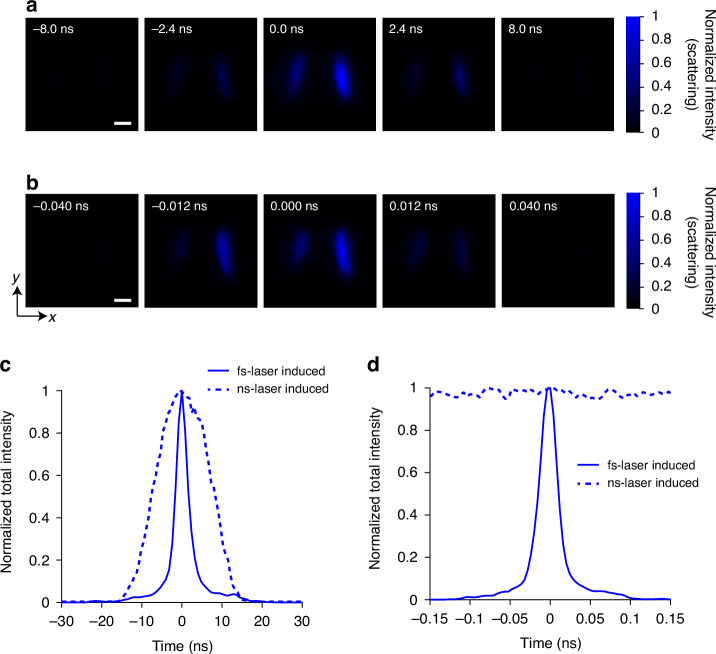
1.25 Gfps and 250 Gfps real-time single-shot imaging of femtosecond laser-excited elastic light scattering (ELS) of soot nanoparticles. **a**, **b** Exemplary snapshots with imaging speeds at 1.25 Gfps and 250 Gfps in **a** and **b**, respectively. Scale bars: 2 mm. Panels **c** and **d** show the comparison of normalized total intensity evolution between femtosecond (solid line) and nanosecond (dashed line) laser-induced ELS signals. **c** Using 1.25 Gfps imaging speed. **d** Using 250 Gfps imaging speed. The temporal spreads of ELS using the fs laser are substantially narrower than those using the ns laser

The red signal (pseudo-colored in Fig. 2b) appears at a location in the flame that resembles region of soot particle formation and therefore it seems natural that it is associated with an LII signal. Yet, while there are approaches using picosecond pulses to induce incandescence^10^, LII is considered unlikely with a short pulse on a femtosecond scale. However, additional spectral measurements, presented in Fig. S3 and compared against the theoretical calculations in Fig. S4, strongly support the finding of laser-induced heating of the soot particles. Note that LIH by femtosecond lasers are different from that of nanosecond lasers, and it is not utilized for soot sizing^25^. In general, the fluence utilized in nanosecond-LII will produce plasma by the same fluence by femtosecond excitation^26^. Therefore, in this work, we have used the femtosecond laser fluence of 0.03 J cm^−2^, which results in moderate heating of the soot particle. The statement that the red signal in Fig. 2b stems from soot particles is supported by elastic light scattering measurements shown in Fig. 3, which spatially overlap with the LIH signals. Here, larger soot particles are much stronger scatterers than molecules, supporting the conclusion that the observed red signal derives from laser heated soot particles, which cool back to initial flame temperature after a few hundred nanoseconds.

Figure 2c illustrates time-resolved integrated intensity plots for PAH-LIF, soot-LIH, and the combination of both optical signals. These plots demonstrate that PAH and soot signals peak at around 0 ns and 20 ns, respectively. At approximately 40 ns, the LIF and LIH signals intersect, with LIF reaching nearly half of its peak intensity, and LIH achieving a similar level. Around 60 ns, LIH and combined signals (LIF + LIH) converge and become equal, since PAH LIF intensity reaches zero. A map of the resulting lifetimes is shown in Fig. 2d and clearly reveals a separation between both of the signals. These findings provide insights into the interactions between PAH molecules and soot particles distributed in a 2D plane, probed using fsLS-CUP. Notably, we observe the formation of very small PAHs in the flame’s central region, while soot covers the PAHs and extends further into the oxidation zone, exhibiting higher heat release. Our observations are consistent with time integrated yet spatially resolved PAH-LIF and soot-LII detection performed in laminar sooty diffusion flames in^5^.

### Ultrafast observation of elastic light scattering

Figure 3a, b illustrates the real-time monitoring of femtosecond laser-soot interactions in terms of ELS intensity^27,28^ generated by a 400 nm femtosecond laser. The spatiotemporal maps of the ELS signal are evident in a sequence of 2D snapshots. The shorter and instantaneous time of the ELS signal, compared to signals from soot-LIH and PAH-LIF (Fig. 2), is primarily due to the inherent characteristics of the laser pulse. The imaging speed is 1.25 Gfps in Fig. 3a and 250 Gfps in Fig. 3b, with a frame interval of 0.8 ns and 4 ps, respectively. The complete sequences can be found in Supplementary Movies S2 and S3. To understand the intensity variations in Fig. 3a, b, we need to consider the formation and evolution mechanisms of soot in the flame. At the combustion initiation stage, hydrocarbon fuels decompose into small radicals, which polymerize into larger hydrocarbon radicals and, under fuel-rich conditions, form PAHs, the precursors of soot^21^. The sizes of the radicals and the PAHs are normally at sub-nanometer scale. PAHs further grow up through more reactions and nucleation into incipient soot particles with the characteristic size of a few nanometers^19^. As combustion progresses, these incipient soot particles grow into larger primary soot particles (10–100 nm) through surface reactions and coagulation. Soot evolves into fractal soot through aggregation^28^. Results in Fig. 3a, b show that at the initiation phase, weak scattered light intensities result from small radicals and PAHs. As we move higher in the burner, incipient soot particles form, and more prominent scattered signals appear. With increasing height above the burner (HAB) primary soot forms through surface growth, increasing both volume fraction and mean size. During soot evolution, the refractive index of soot particles increases^29^, significantly enhancing scattering signals. In conclusion, the scattered light intensities at different HABs reflect the relative distributions of soot particles with different sizes. It’s important to highlight that the band-pass filter used for ELS detection has a narrow transmission bandwidth of only 5 nm, effectively eliminating any fluorescence signal emanating from PAHs (see Fig. S2). Additionally, previous studies have reported that scattering from PAH molecules is also negligible^30^.

The normalized total intensity of ELS reaches its minimum at 6 ns in Fig. 3a and 0.030 ns in Fig. 3b. Figure 3c shows a comparison between femtosecond laser-induced and nanosecond laser-induced ELS. While fs-laser-induced scattering signal has a full width at half maximum (FWHM) of 3.2 ns, corresponding to an effective frame rate of 0.31 Gfps, the ns-laser-induced scattering signal has a FWHM of 15.6 ns. Therefore, it demonstrates the ultrafast capability of our system in comparison to other conventional methods. In Fig. 3d, we tuned up fsLS-CUP’s imaging speed to 250 Gfps and reached more striking evidence in our system’s advantage in resolving temporal information. ELS has a FWHM of only 25 ps for a fs-laser system, corresponding to an effective frame rate of 40 Gfps, while ELS signal remains almost constant for a ns-laser system. Since elastic light scattering is usually considered as an instantaneous event, the intensity plots in Fig. 3c, d essentially measure the temporal responses of the imaging systems. Hence, this showcases the exceptional temporal resolution of our system when contrasted with conventional methods. Using fs laser pulses, Bruno et al^19^. did point measurements with a FWHM of about 12 ps, which is assumed as the time resolution of a streak camera operated at typical conditions. Similarly, most existing measurements are one-dimensional and averaged over multiple laser pulses^18,31^. Therefore, in comparison, fsLS-CUP is advantageous since it provides a two-dimensional, single-shot, and ultrafast single-pulse-based femtosecond laser-induced scattering.

We also notice that the temporal resolution (FWHM of temporal spread) is several times the frame interval (the inverse of the frame rate). For fsLS-CUP at 250 Gfps, this scaling factor is 25 ps/4 ps = 6.25, and for 1.25 Gfps, this factor is 3.2 ns/0.8 ns = 4. This discrepancy is common in most coded optical imaging techniques^32^, such as CUP, compressed ultrafast spectral photography (CUSP)^16^, coded-aperture snapshot spectral imaging (CASSI)^33^ and compressed ultrafast spectral-temporal (CUST)^34^ imaging. The general rule of thumb is that, within the limit of the streak camera’s space resolving capability, the smaller the DMD encoding feature is, the better temporal resolution the system can reach.

It is worth mentioning that for high imaging speeds (e.g. 250 Gfps), the streak tube in the streak camera introduces spatial distortion in the image, which needs to be compensated for. This issue is common in streak cameras^15,35^ operated at high speeds but less at lower speeds (e.g. 1.25 Gfps). This spatial distortion was calibrated by illuminating the fsLS-CUP system through a narrow horizontal slit with an ultra-short laser pulse, as plotted in Fig. S6. A correction step was applied to the raw streak images before reconstruction.

## Discussion

### Gfps-imaging system to capture femtosecond-laser induced processes in combustion

Femtosecond lasers, with their ultra-short pulse durations on the order of femtoseconds, hold immense potential for revolutionizing combustion research and applications. One notable application lies in the field of combustion diagnostics, where femtosecond lasers can be employed for precise and rapid probing of combustion processes. The extremely short pulses enable high-temporal-resolution measurements, allowing researchers to capture intricate details of chemical reactions and flame dynamics. Ultrafast femtosecond pump-probe spectroscopy is employed to generate averaged maps of ultrafast phenomena, however, turbulent process like combustion often necessitating single-shot imaging and, soot study requires single-pulse imaging to avoid any changes in soot physical property^36^. We report the development of fsLS-CUP, achieving the world’s fastest single-pulse real-time 2D imaging of femtosecond-laser-flame interactions, boasting an unparalleled imaging speed of up to 250 Gfps and a sequence depth of 220 frames. This speed surpasses the recently reported nanosecond laser single-pulse single-shot approach by 20 times^17^ and conventional high-speed imaging techniques by at least 20,000 times. What sets fsLS-CUP apart is its unique capability—it can capture laser-induced phenomena and enables simultaneous recording of two chemical species. Thanks to its reliance on a streak camera, the imaging speed can be easily adjusted to film femtosecond laser-induced processes as needed without altering the setup.

### Ultrafast simultaneous observation of PAH and soot

The soot inception study elucidates the intricate relationship between PAHs and soot particles, making the simultaneous observation of both species highly valuable^18,21,37^. However, achieving this simultaneous observation using a single nanosecond laser pulse presents a challenge. Soot particles are heated by the laser pulse, necessitating a high laser fluence for excitation. In contrast, PAHs are probed using a much lower laser fluence to generate LIF signals. Consequently, attempting to probe both species with a single laser pulse of high fluence leads to the overwhelming of LIF signals by LIH of soot. An additional challenge for ultrafast PAH studies arises from the need for higher photon counts at increased imaging speeds. An alternative solution involves the use of an extremely short single laser pulse of UV wavelength, exploiting the lifetimes of the two signals with ultrafast detectors. In this study, we leveraged the ultrafast imaging capability of fsLS-CUP, enabling the separation of signals based on their lifetimes - LIF with a lifetime of less than 50 ns and LIH with lifetimes extending to hundreds of nanoseconds. This innovative approach allowed us, for the first time, to observe soot and PAH simultaneously in terms of their lifetimes. Our real-time PAH-LIF and soot-LIH measurements offer the first spatiotemporal insight into the distribution of PAHs and soot in a flame. It’s important to note that we verified the LIF signals at both lower and higher laser fluences sequentially and observed that the distribution of PAH remains similar in both cases. Finally, we have observed that the signal generated through femtosecond laser-induced processes shares close spectral properties and duration with laser-induced incandescence witnessed in soot particles under nanosecond laser pulses.

### Potential applications

The utilization of fsLS-CUP for real-time ultrafast imaging holds the potential to open novel avenues, such as imaging the sizes of PAH molecules through the implementation of femtosecond pulses. This involves incorporating two-channel fluorescence anisotropy at sub-nanosecond intervals within the existing framework^19,38^. The single shots of simultaneous soot and PAH distributions, repeated statistically, can be obtained in turbulent flames, providing a valuable dataset for model validation^39–41^. Moreover, exploring the effects of high laser fluence on soot oxidation and graphitization could be crucial to produce carbon-based nanomaterials^4^. The versatility of fsLS-CUP is evident in its ability to accommodate up to four channels^42^, allowing the simultaneous observation of four distinct species in flames, including those induced by femtosecond filamentation^20^ and flame ignition^43^. Beyond combustion, fsLS-CUP demonstrates its utility in diverse applications, such as planar two-photon fluorescence microscopy^44^, planar fluorescence anisotropy^45^, and selective plane illumination microscopy^46^ of biological samples.

## Materials and methods

### Components of imaging optics

A pair of 2-inch lenses, with focal lengths of *f* = 200 mm (Thorlabs, AC508-200-A-ML) and *f* = 100 mm (Thorlabs, AC508-100-A-ML), images the flame onto a CCD camera (Point Gray, CM3-U3-28S4M-CS) to capture the time-unsheared view. A polarizer, P2 (Thorlabs, LPVISE100-A), selectively allows photons of linearly polarized along the $y$ direction to pass through. A neutral density filter (ND 2.6) and a narrow bandpass spectral filter (SF) are applied to isolate the scattering signal for imaging, while a broadband bandpass spectral filter is employed to select LIF and LIH signals. See Fig. S3 for the measurements of these filters. A 50:50 beamsplitter BS2 (Thorlabs, BS013) separates the time-sheared and time-unsheared views. For recording time-sheared views, a stereoscopic lens assembly (SL) with a numerical aperture (NA) of 0.5 (Olympus, MV PLAPO 2XC), along with an *f* = 200 mm lens RL1 (Thorlabs, AC508-150-A-ML) relays the image to the digital micro-mirror device DMD (Texas Instruments, LightCrafter 3000) for spatial encoding. Two complementarily masked images, encoded by *C*_1_ and *C*_2_, are then collected and relayed to the streak camera (Hamamatsu, C7700) by the same SL and two 1-inch lenses (RL3 and RL4) with *f* = 200 mm (Thorlabs, AC254-200-A-ML). Two prism mirrors (Thorlabs, MRA12-E02) and a knife-edge right-angle prism mirror KRPM (OptoSigma, KRPB4-15-550) assist in mapping these two images to two sides of the streak camera’s entrance without overlap and cropping the field-of-view. All components are labeled in Fig. 1a.

### Supplementary information

Supplementary Information

Supplementary Movie 1

Supplementary Movie 2

Supplementary Movie 3

## Data Availability

The data that support the findings of this study are available from the corresponding author on reasonable request.

## Code availability

The reconstruction algorithm is described in detail in Supplementary Information. We have opted not to make the computer code publicly available because the code is proprietary and used for other projects.

Supplementary information accompanies the manuscript on the Light: Science & Applications website (http://www.nature.com/lsa).
