# Supplementary material for: Single-pulse ultrafast real-time simultaneous planar imaging of femtosecond laser-nanoparticle dynamics in flames
Source: Light Sci Appl. 2024 Aug 29;13:221. doi: 10.1038/s41377-024-01588-x (PMC11362337; doi:10.1038/s41377-024-01588-x)
Supplement: Supplementary file 1 — Supplementary Information [file 41377_2024_1588_MOESM1_ESM.pdf]

Supplementary Information for

Single-pulse ultrafast real-time simultaneous planar imaging of femtosecond laser-nanoparticle dynamics in flames

Yogeshwar Nath Mishra[1,2,3,‡], Peng Wang[1,‡], Florian J. Bauer[3,‡] , Murthy S. Gudipati[2], and Lihong V. Wang[1,*]

[1]Caltech Optical Imaging Laboratory, Andrew and Peggy Cheng Department of Medical Engineering, Department of Electrical Engineering, California Institute of Technology, 1200 East California Boulevard, Mail Code 138-78, Pasadena, CA 91125, USA

[2]Science Division, NASA-Jet Propulsion Laboratory, California Institute of Technology, 4800 Oak Grove Drive, Pasadena, CA 91109, USA

[3]Lehrstuhl für Technische Thermodynamik (LTT) and Erlangen Graduate School in Advanced Optical Technologies (SAOT), Friedrich-Alexander-Universität Erlangen-Nürnberg (FAU), 91058 Erlangen, Germany

*Corresponding author: LVW@caltech.edu

‡These authors contributed equally to this work

1. Hardware of fsLS–CUP

A. Femtosecond laser sheet

A Ti-Sapphire femtosecond laser (Coherent, Libra HE) generated 800 nm, 500 Hz pulses with a pulse energy of 4 mJ. A BBO crystal is used to convert this femtosecond pulse to the second-harmonic pulse with the center wavelength of ~ 400 nm, a pulse energy of 0.7 mJ, and linear polarization along the y direction. A shortpass filter (Omega Filters, RPE450SP) is used to block the 800 nm light. A laser sheet, 10 mm in height (along the y-axis) and 250 μm in width (along the z-axis), is formed by using a cylindrical lens with $f = 500$ mm (Thorlabs, LJ1144RM). The laser fluence can be easily adjusted using a half-waveplate (Thorlabs, AHWP10M-600) in conjunction with a linear polarizer (Thorlabs, GL15).

B. Digital micro-mirror device (DMD)

The DMD, comprising an array of highly reflective pixels, is used for spatial encoding. These pixels can be electronically controlled to turn in opposite directions, forming two complimentarily patterned imaging paths[2,3]. The DMD displays only one pseudo-random binary mask for single-shot imaging. The raw image of an example computer-generated encoding mask is plotted in Fig. S1. The left and right segments represent two complimentary binary patterns from the two DMD-reflected beam paths. Note that this image was captured while the fsLS-CUP imaging system was flood-illuminated by a continuous-wave white flashlight, and the sweeping voltage in the streak camera was turned off.

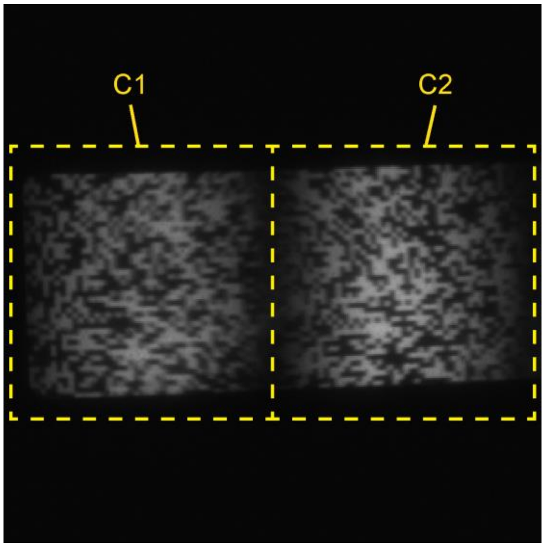

Fig. S1. Raw image of the calibrated encoding pattern displayed by the DMD and captured by the streak camera without the sweeping voltage. The left and right blocks represent complementary masks from the two beam paths reflected by the DMD.

C. Streak camera

In a typical streak camera [1], when photons from a transient event bombard the photocathode, photoelectrons are generated. These electrons are then accelerated by a high-voltage anode. Subsequently, an ultrafast sweeping voltage is applied in a direction orthogonal to the direction that electrons travel, inducing time-dependent shearing. Electrons arriving at a later time (e.g., t_3 in Fig. S2) are deflected to locations farther away than those from an earlier time (e.g., t_1 in Fig. S2). Following this, a phosphor screen converts the 2D map of electrons into an image of photons. An image intensifier is a necessary next step to amplify the signals, compensating for the low efficiencies in the image formation pipeline of the streak camera. Finally, an imaging lens assembly projects the image onto a CMOS sensor to complete the acquisition.

The bottom left inset gives an example of the applied voltage in the streak tube. Essentially, the imaging speed of the streak camera is solely determined by the slope of this voltage ramp. The steeper the voltage ramp, the faster the frame rate of the fsLS-CUP system. For instance, an imaging speed of 100 Gfps requires a voltage ramp 10 times faster than an imaging speed of 10 Gfps. To calculate the imaging speed, we need to know the total time electrons take to sweep across the entire sensor, typically provided by the manufacturer with multiple options. For our streak camera model, the total number of sensor pixels in the sweeping direction is 1000. If a total time window of 5 ns is selected, then the photons between neighboring pixels are delayed by 5 ns / 1000 = 5 ps, suggesting a frame rate of 1 / 5 ps = 200 Gfps. If a binning of 2×2 pixels is applied, the frame rate reduces to 100 Gfps.

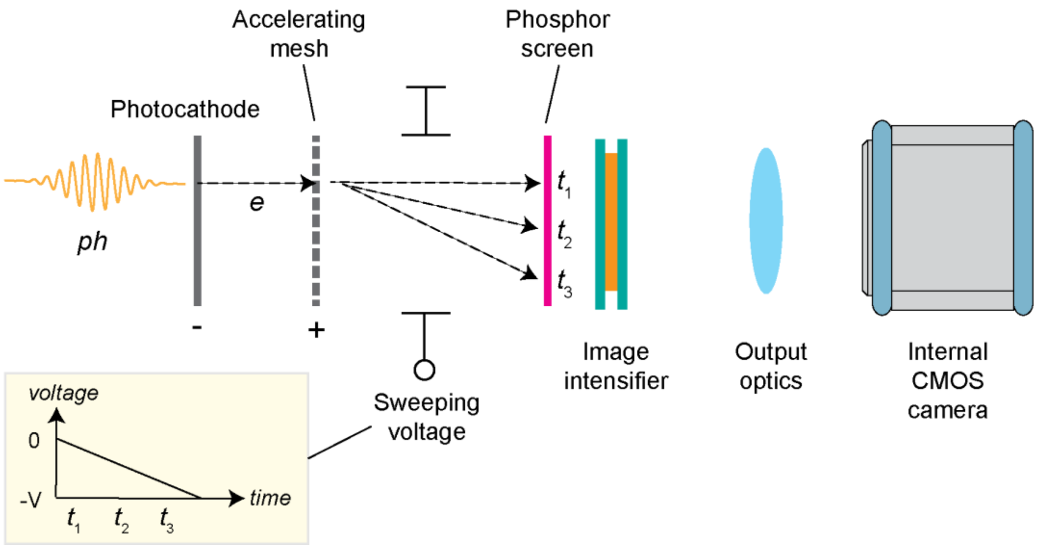

Fig. S2. Schematic of a streak camera. Electrons originated from photons of at different times t_1, t_2 and t_3, are deflected to distinct vertical locations by an ultrafast sweeping voltage (bottom left inset).

D. Spectrum filters

We measured the transmission spectra of two bandpass filters used in fsLS-CUP imaging. The first is a narrow bandpass filter (Semrock, FF01-403/5-25) with a center wavelength of 403 nm and a bandwidth of 5 nm. This filter selectively allows the second harmonic of the femtosecond laser to be imaged, capturing the elastic light scattering signal from the flame. The second is a broad bandpass filter (Semrock, FF01-460/60-25), centered at 460 nm with a bandwidth of 60 nm, which covers the LIF and LIH signals while effectively suppressing the excitation light.

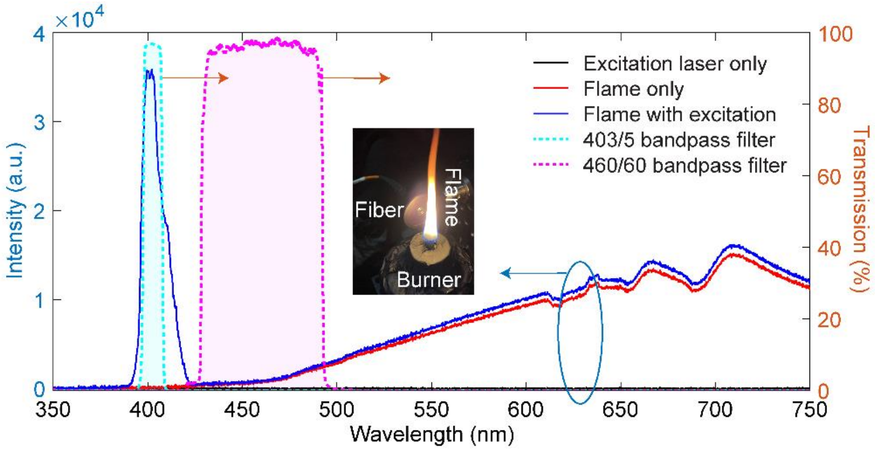

Fig. S3. Spectrometer measurements. Solid lines on the left axis represent measurements near the interaction zone of flame and femtosecond laser sheet. The black solid line indicates the scenario that there is only laser sheet and no flame. The red solid line denoted the case that there is only the flame and no laser sheet. The blue solid line denotes that both laser sheet and flame interact. Dashed lines on the right axis depict the transmission spectra of the filters used in the experiments. Cyan dashed line: narrow bandpass filter with a 403 nm center wavelength and 5 nm bandwidth, utilized for imaging scattering. Magenta dashed line: a broad bandpass filter with a 460 nm center wavelength and 60 nm bandwidth, used for imaging LIF and LIH signals simultaneously. Inset: a photograph capturing the measurement of the flame spectrum, with and without laser excitation, using a fiber spectrometer.

2. Spectral measurements of the flame

To attribute the detected 2D lifetime signals to specific phenomena and avoid crosstalk from multiple signals, additional spectral measurements were conducted. A photograph of the measurement setup is shown as inset in Fig. S3. A fiber spectrometer (Avantes, ULS3648-USB2) is used with an acquisition time of 100 ms. A measurement with only the fs laser pulse in ambient air showed minimal signals (black solid line), indicating the absence of plasma emissions or filamentation. The sheer spectra of the flame luminosity without any laser excitation (red solid line) matches the incandescence signal of heated soot particles. The corrugation of the spectra beyond 600 nm result from the transmission characteristics of the optical components, such as optical fiber, as the experimental setup is not corrected for spectral efficiency. Upon exciting the flame species with the laser pulse (blue solid line), two phenomena are observed: first, the elastic light scattering of the laser pulse on the soot particles produces a strong peak around the laser wavelength of 400 nm. Second, the intensity of the entire spectral range slightly increases, possibly due to the superposition of enhanced thermal radiation from the heating of the soot particles. A LIF signal of differently sized aromatics structures would not lead to such a broadband enhancement, as shown by Bejaoui et al [4]. To support our finding of laser heating, theoretical soot particle emission spectra (see Fig. S4) were calculated based on a typical flame temperature of 1800 K (blue line) compared to a scenario where 50 laser pulses within the acquisition window were added, resulting in an approximate peak temperature of 3000 K (red line). Here, the particle size d_p was 30 nm, and the absorption function $E(m)$ = 0.3 with Boltzmann's constant k_B, Planck's constant h and the speed of light c [5].

$$S = \frac{4\pi d_{\mathrm{p}} E(m)}{\lambda} \frac{2\pi h c^2}{\lambda^5} \frac{1}{e^{\frac{hc}{(\lambda k_B T)}} - 1} \qquad \text{(S1)}$$

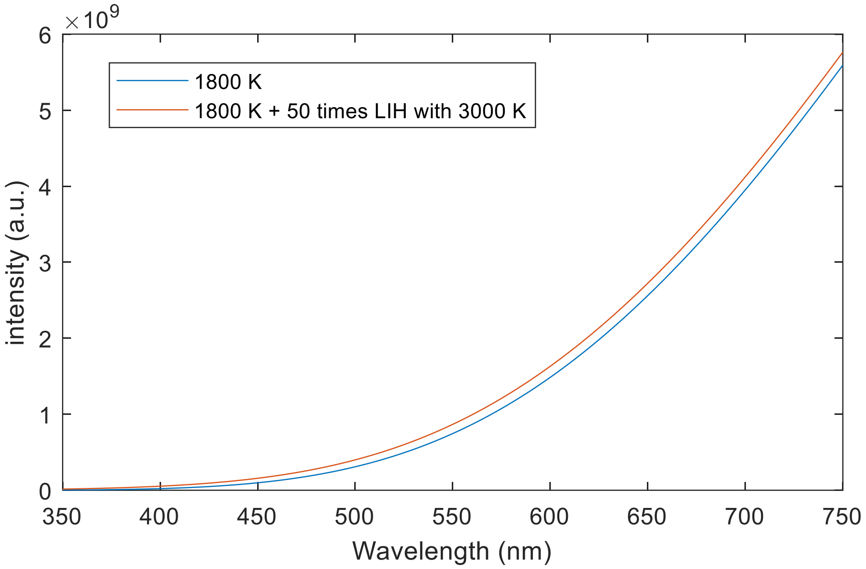

Fig. S4. Theoretically calculated emission spectra of soot particles. The two solid lines show the emission spectra of soot particles for two temperatures cases: 1800 K alone, and 1800 K combined with 50 instances of 3000 K, serving as the LIH signal within the same wavelength range as the spectral measurements of Fig. S3.

3. Data processing in fsLS-CUP

A. Ultrafast movie reconstruction

The image formation in fsLS-CUP can be modeled by

$$E = \boldsymbol{O}I. \tag{S2}$$

In Equation (S2), $I = I(x, y, t)$ is the unknown transient scene to solve. E combines all three images by

$$E = \begin{bmatrix} E_0 \\ \alpha_1 E_1 \\ \alpha_2 E_2 \end{bmatrix}, \tag{S3}$$

in which E_0 represents the time-unsheared view, captured by a conventional CCD camera; E_1 and E_2 represent two complimentarily masked time-sheared view taken by the streak camera. Additionally, α_1 and α_2 are the weighting factors to balance the varying transmissions among different viewing channels.

In Equation (S2), $\boldsymbol{O}$ is a collection of all operators in the imaging pipeline, expressed by [3]

$$\boldsymbol{O} = \begin{bmatrix} \boldsymbol{TF_0} \\ \boldsymbol{TSD_1F_1C}_{[10]} \\ \boldsymbol{TSD_2F_2C}_{[01]} \end{bmatrix}, \tag{S4}$$

In Equation (S4), $\boldsymbol{C}_{[10]}$ and $\boldsymbol{C}_{[10]}$ represent a pair of complementary spatial encoding masks. $\boldsymbol{F}_j$ $(j = 0, 1, 2)$ describes spatial low-pass filtering due to the optics. Image distortion is considered by using $\boldsymbol{D}_j$ $(j = 1, 2)$. $\boldsymbol{S}$ represents temporal shearing and $\boldsymbol{T}$ represents spatiotemporal integration. $\boldsymbol{C}_{[10]}$ and $\boldsymbol{C}_{[10]}$ are recorded by flood illuminating the imaging module with a green laser diode. $\boldsymbol{D}_j$ are extracted via a custom-built algorithm [6].

Regularization is utilized here to recovering I from E and $\boldsymbol{O}$, equivalent to solving the following optimization problem

$$\hat{I} = \mathrm{argmin}_I \left\{ \frac{1}{2} \| E - \boldsymbol{O}I \|_2^2 + \beta \Phi(I) \right\}. \tag{S5}$$

Equation (S5) minimizes the difference between the reconstructed and measured data (first term), confined by a regularizer (second term) that conforms sparsity in the search for the optimal solution. These two terms are balanced by β. Here, $\Phi = \Phi_{\mathrm{TV}}$ contains total variation in the spatiotemporal domains [3]. Two-step iterative shrinkage/thresholding algorithm (TwIST) is employed to solve Equation (S5) [7].

B. Lifetime extraction

Lifetimes of both LIF and LIH signals are determined by fitting their intensity decay curves over time. Two representative examples are shown in Fig. 2c in Main Text. The fitting procedure relies on a least squares optimization, assuming a monoexponential decay function given by $I = C_1 * \exp(-C_2 * t)$, where the decay time (τ) is extracted from $\tau = 1/C_2$. For the regression analysis, only data points that fall above 10% of the noise level and below 90% of the maximum signal intensity are considered. It is worth

noting that both LIF and low fluence LII (LIH, in our case) signals are known to exhibit monoexponential decay [8].

C. Separation of LIF and LIH from single acquisition

This section explains how Fig. 2b is generated. The reconstructed movie (Fig. 2a and the left panel in Supplementary Movie 1) contains both LIF and LIH signals, acquired simultaneously. After generating the 2D lifetime map (Fig. 2d) by scanning each spatial point in the reconstructed movie, we distinguish LIF and LIH signals by applying a threshold on lifetime – set at 38 ns in this work. We chose LIF mask threshold based on the following: the PAHs with two to five rings, categorized as small and medium, exhibit diverse fluorescence lifetimes, ranging from a few hundred picoseconds to several hundred nanoseconds. Notably, PAHs that emit strong fluorescence are linked to shorter fluorescence lifetimes. The flame environment is anticipated to contain a broad spectrum of PAHs. Only those PAHs with elevated fluorescence quantum yield, leading to shorter lifetimes, will play a role in LIF [9,10]. This operation produces two spatial masks: one for LIF (Fig. S5a) and one for LIH (Fig. S5d). These masks are then applied to the original reconstruction results (Fig. 2a and the left panel in Supplementary Movie 1) so that LIF and LIH signals can be pseudo-colored for visualization (see Figs. S5b and S5e). A version combining both signals is shown in both Fig. 2b and the right panel in Supplementary Movie 1. Finally, separate lifetime maps for LIF and LIH signals are calculated (see Figs. S5c and S5f).

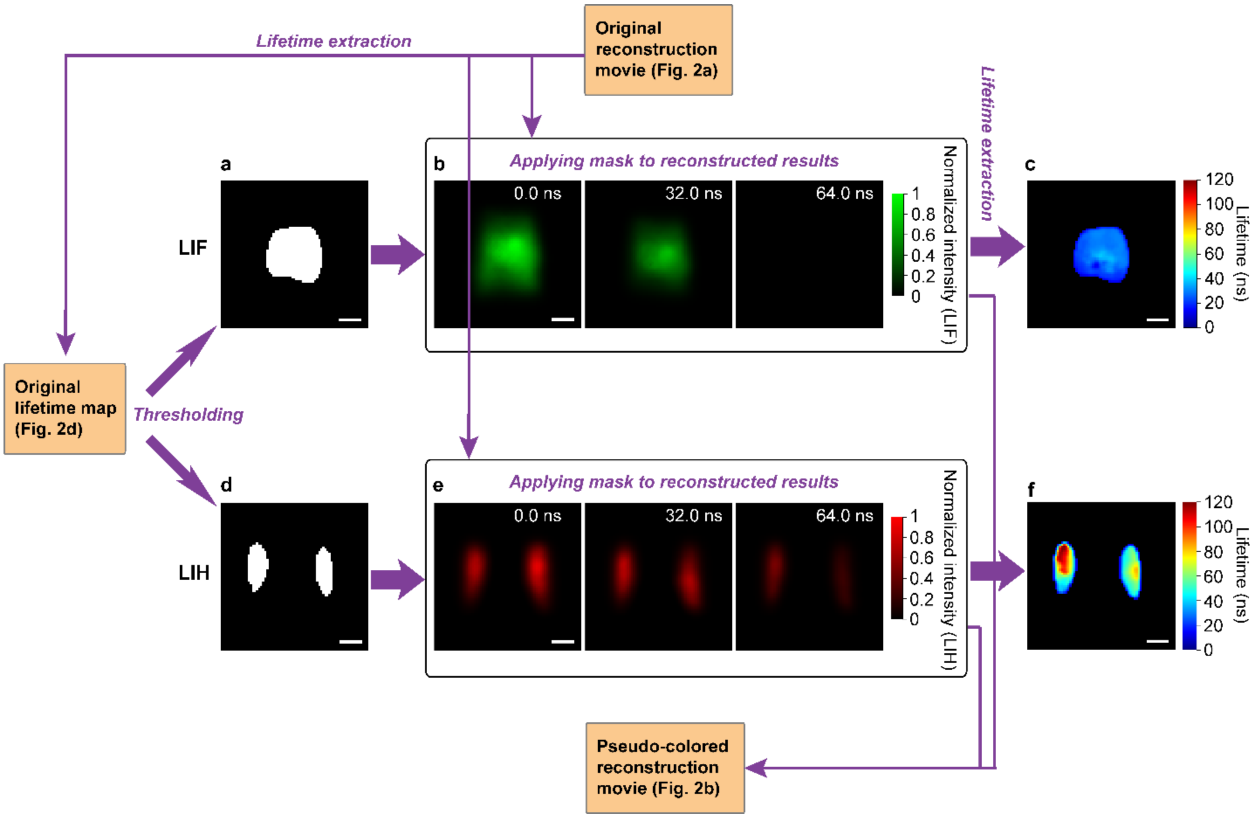

Fig. S5. Separation of LIF and LIH. A threshold of 38 ns in the original lifetime map (Fig. 2d) is used to separate LIF and LIH signals. (**a**) and (**d**) Spatial masks for LIF and LIH after thresholding, respectively. (**b**) and (**e**) Pseudo-colored movies of LIF and LIH dynamics after applying their corresponding masks to the original reconstruction (Fig. 2a). (**c**) and (**f**) Lifetime maps of LIF and LIH by processing their individual spatiotemporal dynamics in **b** and **e**, respectively. Scale bars: 2 mm.

4. Distortion calibration of streak camera

The spatial slant distortion of the streak camera at 250 Gfps was calibrated. A femtosecond laser pulse flood-illuminated the fsLS-CUP imaging system, and a narrow horizontal line was displayed on the DMD, acting as a slit. Two images were captured on the streak camera: one without ultrafast shearing (showing a static image of the slit) and one with 250 Gfps shearing. The differences in vertical positions of the points between these two images of the slit give the calibration curve, as plotted in Fig. S6 below. The raw streak image is corrected by subtracting these differences in the number of pixels at the corresponding horizontal locations.

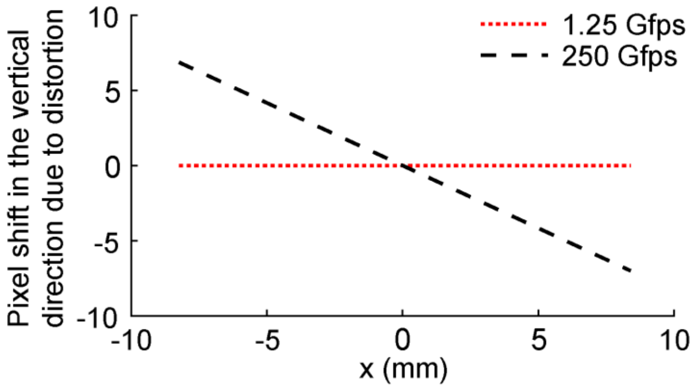

Fig. S6. Calibration of spatial slant distortion for the streak camera at both 1.25 Gfps and 250 Gfps.

REFERENCES

1 *Guide to Streak Cameras*, <https://www.hamamatsu.com/resources/pdf/sys/SHSS0006E_STREAK.pdf> (2008).

2 Gao, L., Liang, J., Li, C. & Wang, L. V. Single-shot compressed ultrafast photography at one hundred billion frames per second. *Nature* **516**, 74-77 (2014).

3 Liang, J. *et al.* Single-shot real-time video recording of a photonic Mach cone induced by a scattered light pulse. *Science Advances* **3**, e1601814 (2017).

4 Bejaoui, S., Mercier, X., Desgroux, P. & Therssen, E. Laser induced fluorescence spectroscopy of aromatic species produced in atmospheric sooting flames using UV and visible excitation wavelengths. *Combustion and Flame* **161**, 2479-2491 (2014).

5 Michelsen, H. A., Schulz, C., Smallwood, G. J. & Will, S. Laser-induced incandescence: Particulate diagnostics for combustion, atmospheric, and industrial applications. *Progress in Energy and Combustion Science* **51**, 2-48 (2015).

6 Wang, P., Liang, J. & Wang, L. V. Single-shot ultrafast imaging attaining 70 trillion frames per second. *Nature Communications* **11**, 2091 (2020).

7 Bioucas-Dias, J. M. & Figueiredo, M. A. A new TwIST: two-step iterative shrinkage/thresholding algorithms for image restoration. *IEEE Transactions on Image processing* **16**, 2992-3004 (2007).

8 Zimmermann, F. P., Koban, W., Roth, C. M., Herten, D.-P. & Schulz, C. Fluorescence lifetime of gas-phase toluene at elevated temperatures. *Chemical Physics Letters* **426**, 248-251 (2006).

9 Burdett, J. J., Müller, A. M., Gosztola, D. & Bardeen, C. J. Excited state dynamics in solid and monomeric tetracene: The roles of superradiance and exciton fission. *The Journal of Chemical Physics* **133**, 144506 (2010).

10 Whitaker, T. J. & Bushaw, B. A. Laser-induced fluorescence analysis of vapor-phase pyrene. *The Journal of Physical Chemistry* **85**, 2180-2182 (1981).

Captions of Supplementary Movies

Movie S1. Simultaneous 1.25 Gfps real-time imaging of both laser-induced fluorescence (LIF) and laser-induced heating (LIH) in a kerosene flame. Both LIF and LIH are excited by a single 0.03 J cm^{-2} 400 nm femtosecond pulse. There are 220 frames acquired in single shot. The left panel is the reconstructed movie containing both LIF and LIH signals plotted in the grayscale colormap. The right panel is the combined movie of LIF and LIH signals reconstructed separately. Pseudo color is used to distinguish LIF (green) and LIH (red).

Movie S2. Real-time 1.25 Gfps imaging of elastic light scattering in a kerosene flame, excited by a single 0.03 J cm^{-2} 400 nm femtosecond pulse. A narrow bandpass filter is used to select the scattering signal. The interval between neighboring frames is 0.8 ns.

Movie S3. Real-time 250 Gfps imaging of elastic light scattering in a kerosene flame, excited by a single 0.03 J cm^{-2} 400 nm femtosecond pulse. A narrow bandpass filter is used to select the scattering signal. The interval between neighboring frames is 4 ps.
